# Rapidly Resolving Nonalcoholic Marchiafava-Bignami Disease in the Setting of Malnourishment After Gastric Bypass Surgery

**DOI:** 10.1177/2324709618784318

**Published:** 2018-06-28

**Authors:** Moshe Bachar, Eduard Fatakhov, Christopher Banerjee, Nathan Todnem

**Affiliations:** 1 Augusta University, Augusta, GA, USA

**Keywords:** Marchiafava-Bignami disease, gastric bypass, malnourishment

## Abstract

*Objectives.* We describe a rare case of nonalcoholic Marchiafava-Bignami disease (MBD) in the setting of malnourishment after gastric bypass. *Methods.* A 44-year-old nonalcoholic Caucasian woman with malnutrition after gastric bypass presented with 2 weeks of weakness. The patient acutely became stuporous. Brain magnetic resonance imaging showed lesions in the corpus callosum and internal capsules consistent with MBD. After 10 days of treatment, the patient had resolution of her encephalopathy with return to baseline mental function, with radiological improvement. *Results.* MBD is a rare neurological disorder seen in alcoholics and can rapidly progress to coma or death. Our patient was successfully treated with a regimen typically used in alcoholics. We discuss the relevant literature supporting this regimen. *Conclusions.* This case demonstrates that since the pathophysiological etiology of the disease is malnutrition, MBD patients can be effectively treated with this regimen regardless of the underlying cause.

## Introduction

Marchiafava-Bignami disease (MBD) is a rare disorder strongly associated with chronic alcohol consumption that is characterized by severe neurologic symptoms including dysarthria, impaired or fluctuating consciousness, and quadriparesis and is associated with other neuropsychiatric disorders. This disease was named after Marchiafava and Bignami, who described the symptoms in Italian men who consumed large amounts of Chianti wine.^1^ Heinrich et al^2^ demonstrated that the most common symptoms of MBD included cognitive impairment (seen in all patients), disconnection syndromes, gegenhalten, dysarthria, pyramidal tract symptoms, and seizures. Moreover, males averaging 40 to 60 years of age were most often hospitalized with MBD. With the advent of widely available neuroradiological imaging, the mortality of this disease has decreased from a typically lethal course to about 8%.^2,3^ In this report, we present a patient with clinically severe MBD with complete symptom resolution following treatment. We also review the currently accepted standards of treatment based on previously reported cases.

## Case Report

A 44-year-old Caucasian woman presented to the emergency room with a 2-week history of anorexia, progressive weakness, and difficulty walking. She had a past medical history of obesity, schizophrenia, and bipolar disorder. Her past surgical history was significant for Roux-en-Y gastric bypass surgery 23 years ago. The patient was functionally independent 2 months prior to admission with acute deterioration in the 2 weeks preceding the admission.

Initial examination revealed a markedly pale and malnourished woman with a body mass index of 14.9 who had lost 8 kg in the preceding 4 months. She had tachycardia with mild epigastric tenderness. She was awake and oriented to person, place, and time. Her pupils were equal, round, reactive to light, and her cranial nerve function was normal. Her strength was 4/5 in bilateral upper extremities and 3/5 in bilateral lower extremities, with preserved sensation. Normal reflexes including unsustained ankle clonus were present bilaterally. Laboratory tests on admission revealed the following: white blood cell count 4800/mm^3^ (normal); hemoglobin 10.1 g/dL (mildly low); mean corpuscular volume 111 fL (mildly high); blood glucose 106 mg/dL (normal); albumin 2.2 g/dL (low); prothrombin time 11.3 seconds (normal); total bilirubin 2.3 mg/dL (normal-high); alkaline phosphatase 201 U/L (normal); aspartate aminotransferase 175 U/L (high); alanine aminotransferase 65 U/L (high); ammonia 98 µmol/L (high); folate 16 ng/mL (normal); and vitamin B_12_ 1745 pg/mL (normal). Hepatic ultrasound showed a moderately fatty liver without mass or cirrhosis. Initial therapy consisted of 100 mg thiamine, 1 mg folic acid, multivitamins, and magnesium sulfate. The patient’s home medications, quetiapine and clonazepam, were held. A nasojejunal tube was placed and feeding was started slowly.

**Figure 1.**
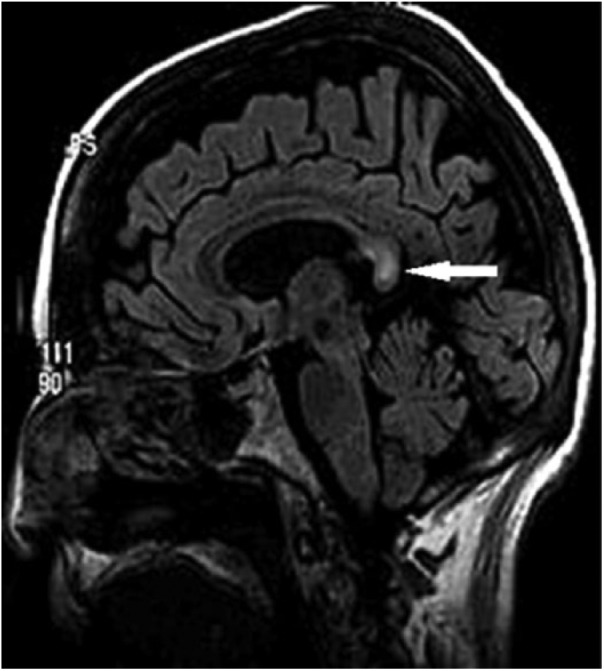
Sagittal fluid attenuated inversion recovery image showing lesion (white arrow) in the splenium of the corpus callosum.

**Figure 2.**
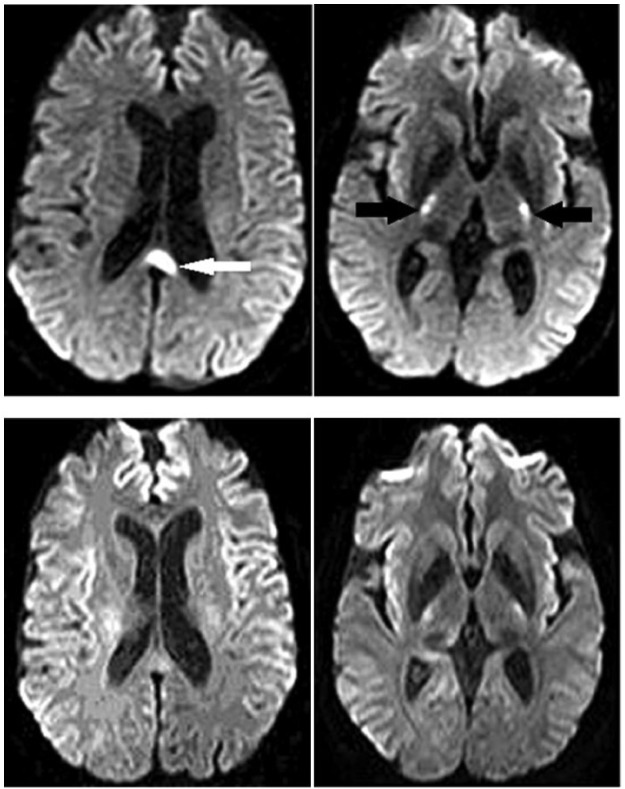
Axial diffusion weighted imaging showing lesions in the splenium (white arrow) and posterior limb of both internal capsules (black arrows). Below are the repeat axial diffusion weighted images obtained 15 days later showing resolution of splenium and internal capsule abnormalities.

The day after admission the patient abruptly became nonverbal and responsive only to pain. She also developed flaccid paralysis of both arms and persistent rhythmic jerking of her legs and feet. These findings are not typical of Wernike or Wernike-Korsakoff syndromes. Refeeding syndrome (with encephalopathic/neurologic manifestations of electrolyte abnormalities) was considered; however, her repeat serum laboratory tests were unremarkable (including sodium of 140, glucose 80, and phosphate 2.9). Additionally, 1-hour electroencephalogram was negative for seizures. Cerebrospinal fluid analysis was unremarkable, ruling out infectious and aseptic meningitis. Brain magnetic resonance imaging (MRI) revealed lesions involving the splenium of the corpus callosum and the posterior limb of both internal capsules (Figures 1 and 2). While extrapontine myelinolysis and hypoglycemic encephalopathy were considered, the aforementioned laboratory values preclude their diagnosis. Atherosclerotic ischemic stroke comes to mind; however, it is inconsistent with this pattern of diffusion restriction. The pattern is most consistent with type B MBD (focal corpus callosum lesions, as opposed to type A with complete callosal involvement), and the patient’s clinical findings match those of MBD. The patient was started on intravenous (IV) methylprednisolone succinate and thiamine, intramuscular cyanocobalamin, and oral pyridoxine. The patient’s course was complicated by a gastrojejunal anastomotic bleeding ulcer and an iatrogenic pneumothorax that eventually necessitated temporary mechanical ventilation. After 10 days of treatment, the patient had complete resolution of her encephalopathy with return to her previous baseline mental function. Repeat brain MRI obtained 15 days from initial scans showed resolution of her splenium and internal capsule defects (Figure 2).

## Discussion

The regimen used to treat this patient is outlined in Table 1 and consisted of the following: thiamine 500 mg IV every 8 hours for 6 doses, then every 24 hours for 5 doses; methylprednisolone 1 g IV every 24 hours for 5 doses; pyridoxine 100 mg PO daily; folic acid 1 mg PO daily; and 2 doses of cyanocobalamin 1 g intramuscular. The dosing regimen for thiamine and folate were chosen partly from the doses used in alcoholics to prevent Wernicke-Korsakoff syndrome.^4,5^ Other B vitamins including pyridoxine and cyanocobalamin have been studied as well, though there is no standard dose. We made the clinical decision to use all 4 given the patient’s known malnutrition and very low negative effects of vitamin administration. The patient was continued on thiamine 100 mg PO daily and folic acid 1 mg PO daily on clinical resolution of the acute disease process.

**Table 1.** An Outline of the Treatment Duration, Dosage, and Medication for Our Patient.

Day	Thiamine	Pyridoxine (Vitamin B_6_)	Methylprednisolone	Cyanocobalamin (Vitamin B_12_)	Folic Acid
1/8-14	100 mg PO q24h				1 mg PO q24h
1/15	100 mg in 50 mL NS IV ×1 and 100 mg PO q24h			1 g IM ×1	1 mg PO q24h
1/16	500 mg in 100 mL NS IV q8h and 100 mg PO q24h				1 mg PO q24h
1/17	500 mg in 100 mL NS IV q8h and 100 mg PO q24h		1 g in 100 mL NS IV q24h	1 g IM ×1	1 mg PO q24h
1/18	500 mg in 100 mL NS IV q24h		1 g in 100 mL NS IV q24h		
1/19	500 mg in 100 mL NS IV q24h		1 g in 100 mL NS IV q24h		
1/20	500 mg in 100 mL NS IV q24h		1 g in 100 mL NS IV q24h		
1/21	500 mg in 100 mL NS IV q24h	100 mg PO q24h	1 g in 100 mL NS IV q24h		1 mg PO q24h
1/22	500 mg in 100 mL NS IV q24h	100 mg PO q24h			1 mg PO q24h
1/23		100 mg PO q24h			1 mg PO q24h
1/24	100 mg in 50 mL NS IV q24h	100 mg PO q24h			1 mg PO q24h
1/25	100 mg in 50 mL NS IV q24h	100 mg PO q24h			1 mg PO q24h
1/26	100 mg in 50 mL NS IV q24h				1 mg PO q24h

Abbreviations: NS, normal saline; IV, intravenous; IM, intramuscular.

The decision was made to use corticosteroids as well. A recent review of literature of 153 MBD (7% which were nonalcoholic) suggests that, while thiamine clearly improves outcomes, steroids did not independently improve outcomes.^6^ As our patient resolved, the treatment course was stopped after 5 doses. Had the patient had edema on her MRIs, resolving edema could have been attributable to steroids; in this absence, it is unclear whether steroids independent of thiamine/vitamins contributed to our patient’s neurological improvement. Additionally, our patient developed a gastrointestinal bleed in the setting of previous bariatric surgery (though correlation is unknown). Steroids are known precipitators of gastrointestinal bleeds, thus caution with the use of steroids in MBD patients is endorsed given the literature showing that steroids do not independently improve outcomes in MBD.

## Conclusion

This case documents the diagnosis of a clinically severe MBD in a nonalcoholic with complete symptom resolution following treatment with thiamine, steroids, and other vitamins. This case also demonstrates that prompt recognition and treatment of this disease may prevent the dramatic and often fatal neurological sequelae that follow. It appears that pathophysiologically MBD arises as a consequence of malnutrition. Thus, this case illustrates that treatment can be generalized across various etiologies of malnutrition in MBD, not just alcoholism.
